# The Genetics of Axonal Transport and Axonal Transport Disorders

**DOI:** 10.1371/journal.pgen.0020124

**Published:** 2006-09-29

**Authors:** Jason E Duncan, Lawrence S. B Goldstein

**Affiliations:** University College London, United Kingdom

## Abstract

Neurons are specialized cells with a complex architecture that includes elaborate dendritic branches and a long, narrow axon that extends from the cell body to the synaptic terminal. The organized transport of essential biological materials throughout the neuron is required to support its growth, function, and viability. In this review, we focus on insights that have emerged from the genetic analysis of long-distance axonal transport between the cell body and the synaptic terminal. We also discuss recent genetic evidence that supports the hypothesis that disruptions in axonal transport may cause or dramatically contribute to neurodegenerative diseases.

## Introduction

The axon of a neuron conducts the transmission of action potentials from the cell body to the synapse. The axon also provides a physical conduit for the transport of essential biological materials between the cell body and the synapse that are required for the function and viability of the neuron. A diverse array of cargoes including membranous organelles, synaptic vesicle precursors, signaling molecules, growth factors, protein complexes, cytoskeletal components, and even the sodium and potassium channels required for action potential propagation are actively transported from their site of synthesis in the cell body through the axoplasm to intracellular target sites in the axon and synapse. Simultaneously, neurotrophic signals are transported from the synapse back to the cell body to monitor the integrity of target innervation. The length of axons in the peripheral nervous system can be in excess of one meter in humans, and even longer in larger animals, making these cells particularly reliant on the efficient and coordinated physical transport of materials through the axons for their function and viability.

The length and narrow caliber of axons coupled with the amount of material that must be transported raises the possibility that this system might exhibit significant vulnerability to perturbation. It has been proposed that disruptions in axonal transport may lead to axonal transport defects that manifest as a number of different neurodegenerative diseases [1]. In this review, we focus on the use of genetics to understand axonal transport, including the identification and functional characterization of components required for axonal transport, and the biological and medical consequences when these functions are compromised.

## Basic Features of the Axonal Transport System

Simplistically, the axonal transport system comprises cargo, motor proteins that power cargo transport, cytoskeletal filaments or “tracks” along which the motors generate force and movement, linker proteins that attach motor proteins to cargo or other cellular structures, and accessory molecules that initiate and regulate transport. Defective axonal transport and neurodegenerative diseases could potentially result from disruptions in any of the components required for axonal transport.

**Figure 1:**
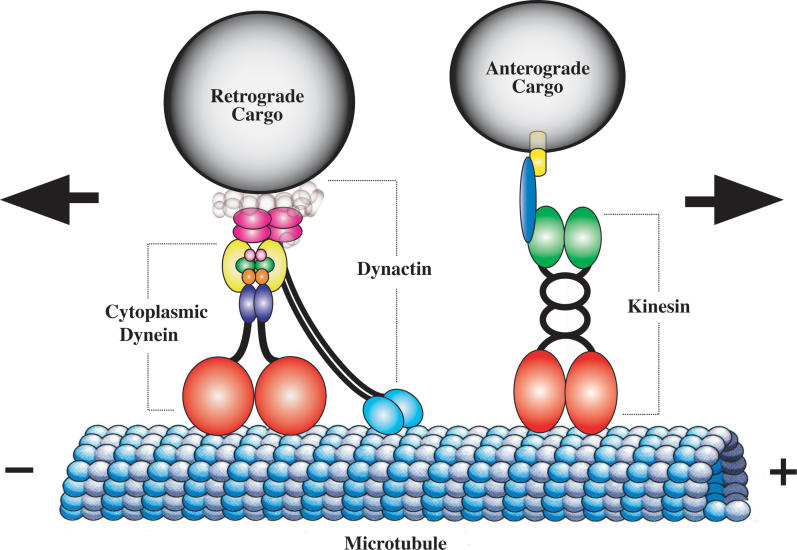
Cytoplasmic Dynein and Kinesin Power Axonal Transport Schematic diagram of the microtubule motor proteins cytoplasmic dynein and kinesin. Cytoplasmic dynein transports cargo in the retrograde direction toward the minus ends of microtubules whereas kinesin transports cargo in the anterograde direction toward the plus ends. Cytoplasmic dynein is a large multimeric protein complex comprising two heavy chain subunits (red) that possess microtubule binding and ATPase activity, two intermediate chains (yellow), two light intermediate chains (indigo), and an assortment of light chains (light pink, green, orange) (reviewed in [7]). Dynactin, a large multisubunit protein complex of comparable size to cytoplasmic dynein, is proposed to link the dynein motor to cargo and/or increases its processivity. The largest dynactin subunit, p150^Glued^ (turquoise), forms an elongated dimer that interacts with the dynein intermediate chain and binds to microtubules via a highly conserved CAP-Gly motif at the tip of globular heads. The dynactin subunit p50 (dark pink) occupies a central position linking p150^Glued^ to cargo. The conventional kinesin holoenzyme, also known as kinesin-1, is a heterotetramer comprising two Khc subunits (red) with microtubule binding and ATPase domains, a central coiled stalk, and a tail domain that interacts with two Klc subunits (green). Klcs may mediate cargo-binding via an intermediate scaffold protein (blue) that binds a cargo transmembrane protein (yellow).

Long-distance transport in the axon is primarily a microtubule-dependent process. The microtubule tracks within an axon possess inherent polarity and are uniformly oriented with the fast-growing (plus) ends projecting toward the synapse and the slow-growing (minus) ends toward the cell body [2]. The motor proteins that power axonal transport on microtubules are members of the kinesin and cytoplasmic dynein superfamilies. Kinesins are generally plus-end–directed motor proteins that transport cargoes such as synaptic vesicle precursors and membranous organelles anterogradely toward the synapse (Figure 1). Cytoplasmic dyneins are minus-end–directed motor proteins that transport cargoes including neurotrophic signals, endosomes, and other organelles and vesicles retrogradely toward the cell body (Figure 1). Retrograde transport may not be exclusive to dyneins, however, as a few kinesins that translocate cargo in the retrograde direction have been identified [3,4]. In mammals, the kinesin superfamily consists of approximately 45 members (KIFs) grouped into 14 subfamilies (reviewed in [5]). Kinesins comprise one to four motor polypeptides called heavy chains that contain a highly conserved motor domain, with ATPase and microtubule-binding regions, and a divergent tail domain. Regulatory and/or accessory subunits, such as the kinesin light chain (Klc), are thought to interact with the tail domain of the kinesin heavy chain (Khc) to confer cargo-binding specificity and regulation (Figure 1) (reviewed in [6]). In contrast to kinesin, the cytoplasmic dynein family in mammals is much smaller, consisting of only two members. Cytoplasmic dynein, however, is a larger and more complex microtubule motor, comprising two dynein heavy chain (Dhc) motor subunits and various intermediate, light intermediate, and light chain (Dlc) subunits (Figure 1) (reviewed in [7]). Cytoplasmic dynein appears to employ a “subunit heterogeneity” approach to support a wide range of essential cellular functions with only a few copies of the cytoplasmic dynein motor peptide and a diverse array of dynein-associated accessory proteins that impart cargo-binding specificity and functional activity [6,8]. Considerable evidence suggests that dynein function is dependent on an equally large protein complex called dynactin, which is proposed to link cytoplasmic dynein to its cargo and/or to increase dynein processivity through an association with microtubules (Figure 1) [9,10].

Based on the kinetics of transport determined from classic pulse-chase labeling experiments, axonal transport is classified as either fast or slow (reviewed in [11,12]). Fast axonal transport occurs in both the retrograde and anterograde directions at a rate of 0.5–10 μm/sec and includes the transport of membrane-bound organelles, mitochondria, neurotransmitters, channel proteins, multivesicular bodies, and endosomes. In contrast, slow axonal transport occurs in the anterograde direction at a rate of 0.01–0.001 μm/sec, considerably slower than fast axonal transport [12]. Cytoskeletal components, such as neurofilaments, tubulin, and actin, as well as proteins such as clathrin and cytosolic enzymes are transported at this slower rate [12]. Current thought is that slow axonal transport is mediated by the same microtubule motors that participate in fast axonal transport, with fast instantaneous transport of cargo interspersed with prolonged pauses [13–15].

## Mutations Disrupting Motor Proteins

Classic studies using extruded squid axoplasm identified kinesin and cytoplasmic dynein as candidate motors required for axonal transport [16–20]. Since then, many different animal model systems have been used to genetically investigate axonal transport mechanisms. Such studies reveal considerable diversity in kinesin function in the axon (Table 1).

**Table 1:**
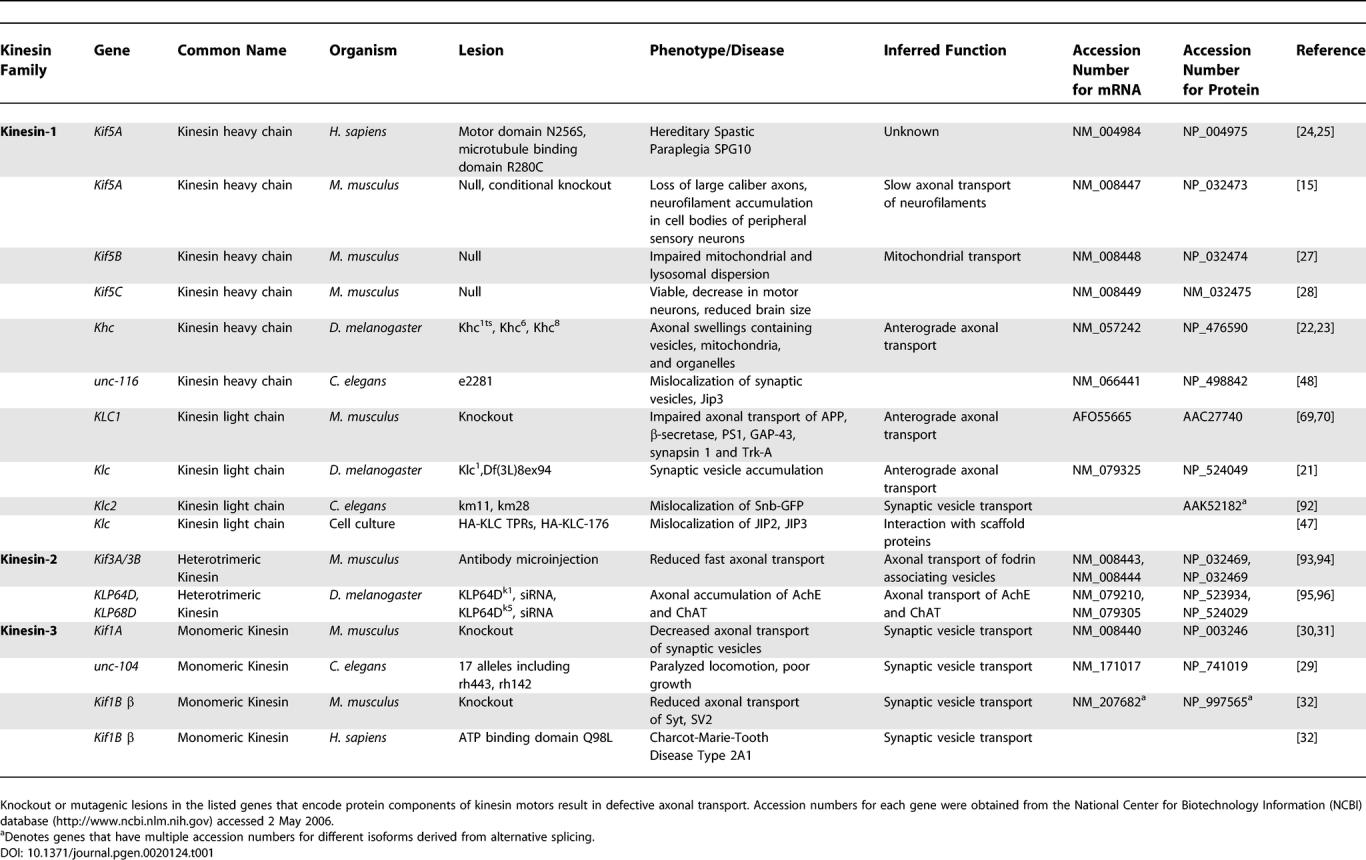
Kinesin Genes Required for Axonal Transport

The requirement for conventional kinesin (Kinesin-1) in axonal transport was revealed in Drosophila melanogaster larvae with lesions in *Khc* and *Klc* genes. These mutants exhibit axonal swellings containing accumulations of transported vesicles, synaptic membranes, and mitochondria [21–23]. Such axonal “organelle jams” are a phenotypic hallmark of compromised axonal transport and result in a posterior paralysis of mutant larvae. Loss of function of the neuronal Kinesin-1 family member KIF5A is linked to the human neurodegenerative disease Hereditary Spastic Paraplegia (HSP) Type 10 (HSP(SPG10)) [24,25]. HSP is a group of clinically heterogeneous neurodegenerative disorders characterized by progressive spasticity and mild weakness of the lower limbs [26]. Although the mechanistic cause of HSP(SPG10) remains unclear, the observation that KIF5A is required for the transport of neurofilaments implies a possible defect in slow axonal transport in the pathogenesis of HSP(SPG10) [15]. The ubiquitous Kinesin-1 family member KIF5B is required for the transport of both mitochondria and lysosomes [27]. Elucidation of a defined cellular role for neuronal-specific Kinesin-1 KIF5C is hindered by its apparent functional redundancy with KIF5A and KIF5B [28].

Members of the Kinesin-3 family, including UNC-104, KIF1A, and KIF1B, are required for the axonal transport of specific membrane-bound organelles such as synaptic vesicle precursors and mitochondria. Mutants of the *unc-104* gene of C. elegans are paralyzed and have fewer synaptic vesicles than wild-type animals [29]. The subcellular distribution of other membrane-bound organelles such as the endoplasmic reticulum, Golgi apparatus, and mitochondria appear normal in these mutants, supporting the idea that the specific role for UNC-104 is in the anterograde transport of synaptic vesicle components [29]. Mice lacking KIF1A, a neuronal-specific homolog of UNC-104, die shortly after birth and suffer marked neuronal degeneration associated with a similar decrease in synaptic vesicle transport and a subsequent reduction in the density of these vesicles in the nerve terminals [30]. Fractionation and immunoisolation experiments revealed that KIF1A associates with a specific subclass of synaptic vesicles containing synaptotagmin, synaptophysin, and Rab3A [31]. KIF1Bβ associates with yet a different subclass of synaptic vesicle components that contain synaptophysin, synaptotagmin, and the synaptic membrane integral protein SV2 [32]. Interestingly, the human neurodegenerative disorder Charcot-Marie-Tooth (CMT) disease Type 2A1, an inherited neuropathy characterized by weakness and atrophy of distal muscles, is linked to a mutation in the ATP binding site of the motor domain of human KIF1Bβ [32]. In a KIF1Bβ knockout, heterozygous mice develop multiple nervous-system abnormalities similar to those observed in UNC-104/KIF1A mutants, including a decrease in the transport of synaptic vesicle proteins and a reduction of these vesicles at the synapse [32].

Together these genetic experiments support the hypothesis that KIFs support various cellular functions by transporting different classes of organelles and vesicles in axons.

Unlike the kinesin superfamily, in which different members of a large superfamily support diverse cellular functions, cytoplasmic dynein comprises an invariant motor subunit with variations in other protein subunits that potentially alter motor function and cargo specificity. Consequently, isolating and interpreting lesions in the cytoplasmic dynein motor has been difficult since dynein is required for multiple functions in the neuron, including axonal transport [33,34]. Nonetheless, in vivo evidence supports a role for cytoplasmic dynein in retrograde axonal transport (Table 2).

**Table 2:**
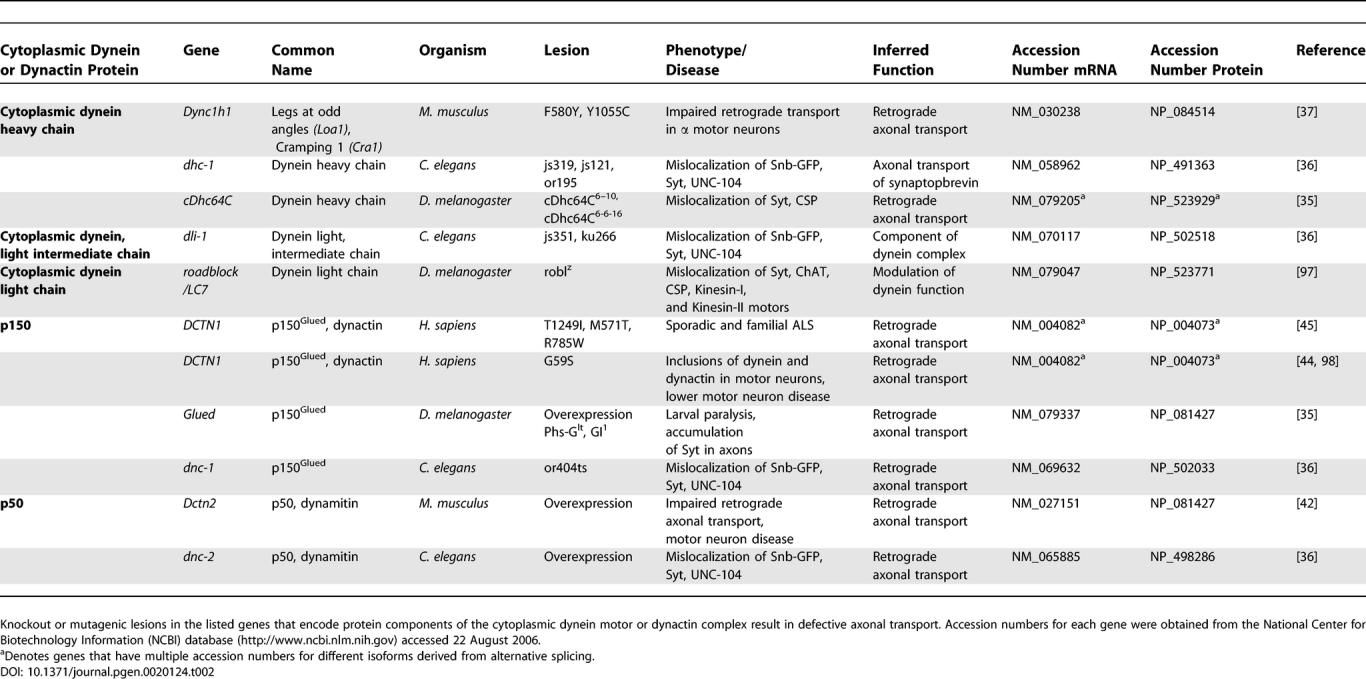
Cytoplasmic Dynein and Dynactin Genes Required for Axonal Transport

Although null mutants die early in development, hypomorphic alleles of the cytoplasmic *Dhc* in *Drosophila* result in larval paralysis with accumulations of synaptic vesicle components in axonal swellings that are indistinguishable from phenotypes observed in Khc mutants [35]. Hypomorphic mutations in both the C. elegans *Dhc* and *Dlc* genes also caused reduced locomotion in animals and ectopic accumulation of the synaptic vesicle components synaptobrevin, synaptotagmin, and the kinesin motor UNC-104 at the terminal ends of mechanosensory processes [36]. Finally, two mutations in the mouse dynein heavy chain gene *(Dync1h1),* *Loa* and *Cra1,* cause progressive motor neuron degeneration in heterozygotes [37]. A marked alteration in the retrograde transport of a fluorescent tetanus toxin tracer was observed in cultured motor neurons isolated from *Loa* homozygous mice [37]. Although mutant forms of the *Dync1h1* gene are ubiquitously expressed in heterozygous mice, the lesions appear to primarily perturb axonal transport in motor neurons, indicating that for unknown reasons, motor neurons are extremely sensitive to alterations in dynein function [37].

## Mutations in Non-Motor Components Disrupt Axonal Transport

Lesions in kinesin and cytoplasmic dynein disrupt critical functions in axonal transport, but factors associated with the motors, such as dynactin, may also be essential for transport (Table 3). Membrane-bound organelles transported in the axon often move bidirectionally, alternating between anterograde and retrograde motion, with net movement in one direction. This suggests that dynein and kinesin are present on the same organelles and their activity is coordinated. One candidate to mediate this coordination is the dynactin complex [38]. Strong genetic interactions have been observed between kinesin, cytoplasmic dynein, and the dynactin complex in *Drosophila* [35]. Dynactin is also required for bidirectional transport of lipid droplets in *Drosophila* embryos and mediates the interaction between kinesin and cytoplasmic dynein in *Xenopus* melanophore cells [39,40]. Consequently, caution must be exercised when interpreting phenotypes associated with mutations in dynactin components because both anterograde and retrograde transport parameters may be affected, as observed in the axonal transport of mitochondria in *Drosophila* p150^Glued^ mutants [41]. In another study, the overexpression of a dominant negative form of dynactin component p150^Glued^ in *Drosophila* caused phenotypes similar to those observed in both Dhc and Khc mutants [35]. Partial loss-of-function of p150^Glued^ or overexpression of p50 dynamitin in C. elegans resulted in ectopic accumulation of synaptic vesicle components [36]. The overexpression of p50 dynamitin disrupts the dynactin complex and inhibits cytoplasmic dynein function, circumventing the difficulty of isolating viable dynein mutants. The targeted overexpression of p50 dynamitin in mouse motor neurons caused an accumulation of synaptophysin and aggregation of neurofilaments in axons, as well as late onset motor neuron degeneration [42]. Although mutant cytoplasmic dynein has yet to be identified as a causative factor of a human neurological disorder, dynactin is directly linked to a number of human neurodegenerative diseases. Lesions in the conserved CAP-Gly microtubule-binding motif of the p150^Glued^ subunit of dynactin have been identified in a family with a heritable form of motor neuron disease. These individuals exhibit weakness in the distal limbs, abnormal accumulations of both cytoplasmic dynein and dynactin in motor neurons, and motor neuron degeneration [43,44]. Three additional lesions in the p150^Glued^ subunit of dynactin have also been identified in patients with amyotrophic lateral sclerosis [45].

**Table 3:**
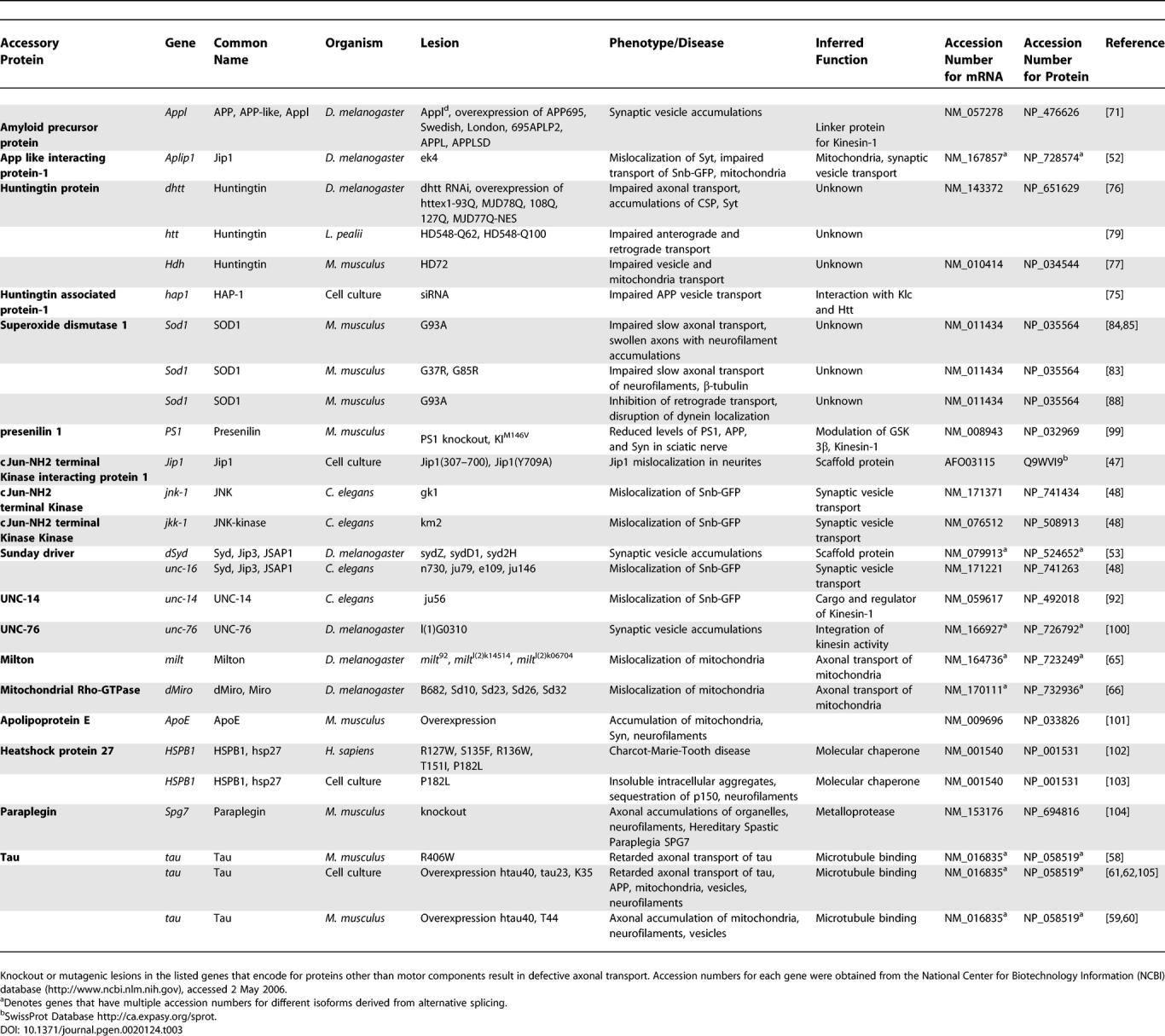
Accessory Genes Required for Axonal Transport

Motor proteins bind to transmembrane proteins on the cargo surface directly, or indirectly, via intermediary scaffold proteins (Figure 1) [6,46]. The cJun NH_2_-terminal kinase (JNK) interacting protein (JIP) group is a class of proteins that may link the kinesin motor to cargo and also act as a scaffold for components of the stress-activated JNK kinase signaling pathway [47]. This implies that the subcellular localization of the JNK signaling complex in the neuron may be regulated by vesicular axonal transport or conversely that kinesin motor activity during axonal transport may itself be regulated via the JNK signaling pathway. In support of the latter, deletion of JNK and JNK kinase results in the mislocalization of synaptic vesicle components in C. elegans [48], although this could be due to a requirement of JNK to regulate microtubule dynamics [49]. The JIP1 and JIP2 proteins are thought to link kinesin with apolipoprotein E receptor 2 (ApoER2) on cargo [50,51]. Aplip1, the *Drosophila* JIP1 homolog, is required in axonal vesicle transport and, curiously, the retrograde transport of mitochondria [52]. Sunday Driver (Syd)/JIP3 was identified in *Drosophila* as a scaffold protein possibly required for the interaction of kinesin with vesicles transported in the axon [53]. Interestingly, Syd/JIP3 is implicated as a transport-dependent positive-injury signal in the response to axonal damage [54].

Another interesting process was recently found in studies of the motor domain of KIF5 which has been suggested to interpret variations in microtubule structure in the neuronal cell body to ensure that cargo is directed into the axon [55]. The mechanism by which this occurs is unclear, but microtubule-associated proteins on the surface of microtubules are probable candidates. The predominant microtubule-associated protein in the axon is tau, which promotes microtubule assembly and stability. Mutations in tau not only impair its ability to bind, stabilize, and assemble microtubules [56,57], but also retard its slow transport in the axon [58]. When tau is overexpressed [59,60] or abnormally phosphorylated [61,62], it forms aggregates that may physically block the fast anterograde transport of mitochondria, neurofilaments, peroxisomes, and vesicles carrying the amyloid precursor protein (APP). The retrograde axonal transport of signaling endosomes that provide neurotrophic support for the neuron may also be blocked and prevented from reaching the cell body [63].

The *Drosophila* proteins Milton and mitochondrial GTPase Miro are also required for the transport of mitochondria [64–66]. Lesions in *Milton* and *Miro* result in the specific failure of mitochondria to be transported anterogradely, and they consequently accumulate in the cell body, although the transport of synaptic vesicles is unaffected.

## Links between Axonal Transport and Human Neurodegenerative Disease

Defects in axonal transport have been indirectly linked to a number of progressive human neurodegenerative diseases including Alzheimer disease (AD), Huntington disease (HD), and amyotrophic lateral sclerosis (ALS). One common feature of these diseases is that the proteins encoded by genes linked to each disease are transported in the axon and can perturb transport when manipulated; presenilin 1 and APP in AD, Cu/Zn superoxide dismutase (SOD1) in ALS, and huntingtin (Htt) in HD. Each disease is characterized by accumulations of these or other proteins within axons, similar to defective axonal transport phenotypes observed in animal models of motor protein mutants.

The pathological hallmarks of AD include neurofibrillary tangles of abnormally phosphorylated tau protein and aggregates of amyloid-β (Aβ) peptide resulting in neuritic plaques in the brain [67]. The transmembrane protein APP, the precursor of potentially neurotoxic Aβ, is transported anterogradely within vesicles in axons by the fast axonal transport system [68]. Interestingly, APP may link the kinesin motor either directly, or indirectly, via the JIP1 scaffold, to a specific class of synaptic vesicles containing synapsin 1, growth-associated protein 43 (GAP-43), along with β-secretase and presenilin 1, two components responsible for processing Aβ from APP [69,70]. Deletion of the APP homolog Appl in *Drosophila* results in defective axonal transport including axonal accumulation phenotypes [71]. Overexpression of human APP causes similar phenotypes that are enhanced by genetic reduction in kinesin and suppressed by genetic reduction in cytoplasmic dynein [71]. These findings suggest that APP plays a central role in the axonal transport of a specific class of vesicle and that disruption in this transport, through lesions in APP or APP-interacting components, may result in axonal blockages, a possible causative factor in the development of AD.

HD is a progressive neurodegenerative disorder caused by expansion of CAG triplet repeats in the coding sequence of the huntingtin gene resulting in an expanded polyglutamine tract (polyQ) in the Htt protein and a toxic gain of function. Interestingly, both Htt and the Huntingtin-associated protein 1 (HAP1) are anterogradely and retrogradely transported in axons [72]. HAP1 interacts with the anterograde motor kinesin via the Klc subunit and is thought to interact with the retrograde motor cytoplasmic dynein through an association with the p150^Glued^ subunit of dynactin [73–75]. Recent studies raise the possibility of a link between axonal transport defects and the onset of HD. In *Drosophila,* both a reduction of Htt protein and the overexpression of proteins containing polyQ repeats result in axonal transport defects [76]. Full-length mutant Htt also impairs vesicular and mitochondrial transport in mouse neurons [77]. Although the mechanism of axonal transport disruption remains unclear, one possibility is that toxic Htt titrates soluble motor protein components into axonal aggregates that physically block transport. One class of vesicle potentially affected are those containing brain-derived neurotrophic factor which would result in loss of neurotrophic support and neuronal toxicity [77,78]. Interestingly, in transport studies performed on extruded squid axoplasm, recombinant Htt fragments with polyQ expansions inhibited fast axonal transport in the absence of aggregate formation [79]. This suggests that polyQ aggregates may not be necessary for axonal transport disruption, but may contribute to or enhance neuronal toxicity. Clearly, a more comprehensive analysis is required to elucidate the mechanism of polyQ toxicity.

Lesions in the ubiquitously expressed enzyme SOD1 are a cause of rare hereditary ALS [80,81]. Mouse models of hereditary ALS have been generated by transgenic expression of mutant SOD1. These animals have impaired slow axonal transport with axonal accumulations of neurofilaments and tubulin [82–85]. Similarly, large axonal swellings with neurofilament accumulations, consistent with a failure in axonal transport, are observed in patients with ALS [86,87]. It has been suggested that SOD1 may specifically inhibit retrograde axonal transport [88]. The potential involvement of cytoplasmic dynein in ALS was further highlighted by the identification of a number of lesions in the motor binding domain of dynactin subunit p150^Glued^ in ALS patients [45]. Additional support comes from the observation that the cytoplasmic dynein mutations *Loa* and *Cra1* revert axonal transport defects of ALS mice, attenuating motor neuron degeneration resulting in delayed onset of disease and extended lifespan [89,90].

## Conclusions and Future Directions

Although a potential link between axonal transport disorders and neurodegenerative disease has been suggested, a number of critical questions remain unanswered. For example, recent evidence indicates that axonal transport is disrupted in mouse models of ALS, HD, and AD long before detectable pathological hallmarks of the disease are observed [77,83,91]. Similarly, comparable pathology may exist early in these human diseases. Yet, it remains unclear whether these changes are causes or consequences of the disease process. Unraveling these issues will require a better understanding of how axonal transport is controlled and which components contribute to the various pathways. In several cases, it is not known whether human mutations represent loss of function or give rise to dominant negative effects, resulting in toxic proteins that titrate or poison axonal transport components. As a result, the effect on axonal transport could be specific and cause the disruption of only a single class of transported material, or nonspecific and reduce or physically block multiple transport pathways through the aggregation of transported cargoes into axonal blockages. It is likely that both mechanisms occur, depending on the nature of the lesion and the motor component involved. Finally, while genetics in model systems will continue to clarify mechanisms, further investigations of heritable neurological disorders in humans may lead to the identification of additional motor proteins or accessory components required for axonal transport. In any event, a more comprehensive understanding of axonal transport may lead to the development of novel therapies for the treatment of neurodegenerative disorders.

## Abbreviations

AchE: acetylcholinesterase
AD: Alzheimer disease
ALS: amyotrophic lateral sclerosis
APP: amyloid precursor protein
ChAT: choline acetyltransferase
CMT: Charcot-Marie-Tooth disease
CSP: cysteine-string protein
Dhc: cytoplasmic dynein heavy chain
Dlc: cytoplasmic dynein light chain
*Dync1h1*: dynein heavy chain gene
GAP-43: growth associated protein 43
GSK 3β: glycogen synthase kinase 3 β
HAP1: Huntingtin-associated protein 1
HD: Huntington disease
HSP: Hereditary Spastic Paraplegia
HSP(SPG 10): Hereditary Spastic Paraplegia Type 10
Htt: huntingtin protein
JIP: JNK interacting protein
JNK: cJun NH_2_-terminal kinase
Khc: kinesin heavy chain
KIFs: kinesin superfamily members
Klc: kinesin light chain
PS1: presenilin-1
Snb-GFP: synaptobrevin-GFP
SOD1: Cu/Zn superoxide dismutase
Syt: synaptotagmin
UNC-104: UNCoordinated-104
